# Enhancing NK cell-mediated cytotoxicity to cisplatin-resistant lung cancer cells via MEK/Erk signaling inhibition

**DOI:** 10.1038/s41598-017-08483-z

**Published:** 2017-08-11

**Authors:** Li Yang, MingJing Shen, Li Jun Xu, Xiaodong Yang, Ying Tsai, Peter C. Keng, Yuhchyau Chen, Soo Ok Lee

**Affiliations:** Department of Radiation Oncology, University of Rochester School of Medicine and Dentistry, Rochester, NY 14642 USA

## Abstract

Major progress has been made clinically in inhibiting the programmed death receptor 1 (PD-1)/PD-L1 interaction to enhance T cell-mediated immune function, yet the effectiveness of anti-PD-L1/PD-1 agents in enhancing natural killer (NK) cell’s function remains largely unknown. Susceptibilities of cisplatin-resistant A549CisR and H157CisR cells vs. parental cells to the cytotoxic action of NK cells were examined. We found cisplatin-resistant cells more resistant to NK cell cytotoxicity than parental cells. There were constitutively higher expressions of PD-L1 in A549CisR and H157CisR cells than in parental cells *in vitro*, as well as in H157CisR cell-derived tumors than H157P cell-derived tumors. In contrast, we observed that the expression of PD-1 in NK cells was induced after co-culture with cisplatin-resistant cells. We also observed increased susceptibility of cisplatin-resistant cells to NK cell cytotoxicity when neutralizing antibody of PD-1 or PD-L1 was added. Further, we found that the NK group 2, member D (NKG2D) ligand levels were lower in A549CisR and H157CisR cells than in parental cells. Meanwhile, we discovered that the MEK/Erk signaling pathway played a significant role in this regulation, and the addition of a MEK/Erk pathway inhibitor significantly enhanced the PD-L1 Ab effect in enhancing NK cell cytotoxicity to cisplatin-resistant cells.

## Introduction

Clinical trials in recent years have demonstrated the effectiveness of immunotherapy for advanced non-small cell lung cancer (NSCLC) after failure of chemotherapies, thus supporting the critical role of improving host immunity in combating NSCLC^1, 2^. Development of multiple anti-programmed death receptor 1 ligand (PD-L1) and anti-PD-1 agents blocking the PD-1/PD-L1 immune checkpoint has been the center of immunotherapeutic approaches^3–5^. However, recent studies showed that tumor cells may escape from T cell-mediated immune reaction^6, 7^ and overexpression of the PD-L1 on cancer cells may be linked to the resistance to anti-cancer therapies^8, 9^.

While major clinical progress has been made in inhibiting the PD-1/PD-L1 interaction to enhance T cell-mediated immune function^6, 7^, research in exploring the effectiveness of anti-PD-L1/PD-1 agents in enhancing natural killer (NK) cells immune function remains scarce^10, 11^. NK cells infiltrate into primary tumor sites at early stages of tumor development^12, 13^. While the adaptive immune cells including (T and B cells) mediate long-lived antigen-specific responses and effective memory^14^, NK cells mediate innate immediate responses by directly killing tumor cells without being sensitized to tumor antigens. Emerging *in vivo* evidence showed that NK cells indeed have an important role in immune defense^15^. Depletion of NK cells leads to enhanced tumor formation in mouse models^16^, also proving the involvement of NK cells in anti-tumor immunity *in vivo*.

In addition to PD-L1/PD-1 interaction, interaction of the activating receptor NKG2D (natural-killer group 2, member D) and its ligands on tumor cells also plays an important role in the NK and T-cell-mediated immune response to tumors. Ligands for NKG2D are rarely detectable on the surface of healthy cells and tissues, but are frequently expressed by tumor cell lines and in tumor tissues^17^. As it has been shown that down-regulation of NKG2D activating ligands, such as UL16 binding protein 1 (ULBP1), ULBP2, ULBP3, and MHC class I chain-related molecules A and B (MICA, and MICB), are key pathways for tumor cells to escape from NK cell-mediated cytotoxic action^17^, NKG2D ligands are also emerging as a potentially important target in immunotherapy^18^.

In this study, we first asked if interactions of PD-L1/PD-1 and NKG2D ligands/NKG2D are important in exerting cytotoxic action of NK cells to lung cancer cells. As we found that cisplatin-resistant cells were more resistant to cytotoxic action of NK cells, we then investigated whether the higher resistance of cisplatin-resistant lung cancer cells to NK cell cytotoxicity than parental cells was due to the alteration of PD-L1/NKG2D ligand levels in cisplatin-resistant lung cancer cells and PD-1/NKG2D levels in NK cells. We then investigated molecular mechanisms that were responsible for the alteration of PD-L1/NKG2D ligand levels in cisplatin-resistant lung cancer cells, and tested whether inhibiting molecular signaling pathways involved in such regulation might enhance the susceptibilities of cisplatin-resistant lung cancer cells to NK cell cytotoxicity.

## Results

### Cisplatin-resistant cells were resistant to NK cell-mediated cytotoxicity compared to parental cells

We have developed two cisplatin-resistant NSCLC cell lines, A549CisR and H157CisR, by treating A549P and H157P cells with increasing concentrations of cisplatin over 6 months. These cells showed IC_50_ values 5–6 times higher than parental cells (data published previously)^19^. NK cytotoxicities of parental vs. cisplatin-resistant cells were investigated using two NK cell sources for the experiments. The established NK92 cell line was known to exhibit high NK cytotoxicity and has been widely used in *in vitro* and in mouse studies^20, 21^. The primary NK cells isolated from the peripheral blood mononuclear cells (PBMCs) had the purity of higher than 90% of CD56+ CD3− NK cell markers, which had been confirmed by flow cytometric analyses (data not shown). We applied two different assays to monitor NK cell mediated cytotoxicity: the lactate dehydrogenase (LDH) release-based NK cytotoxicity test^22–25^, and the colony formation assay^26^.

**Figure 1:**
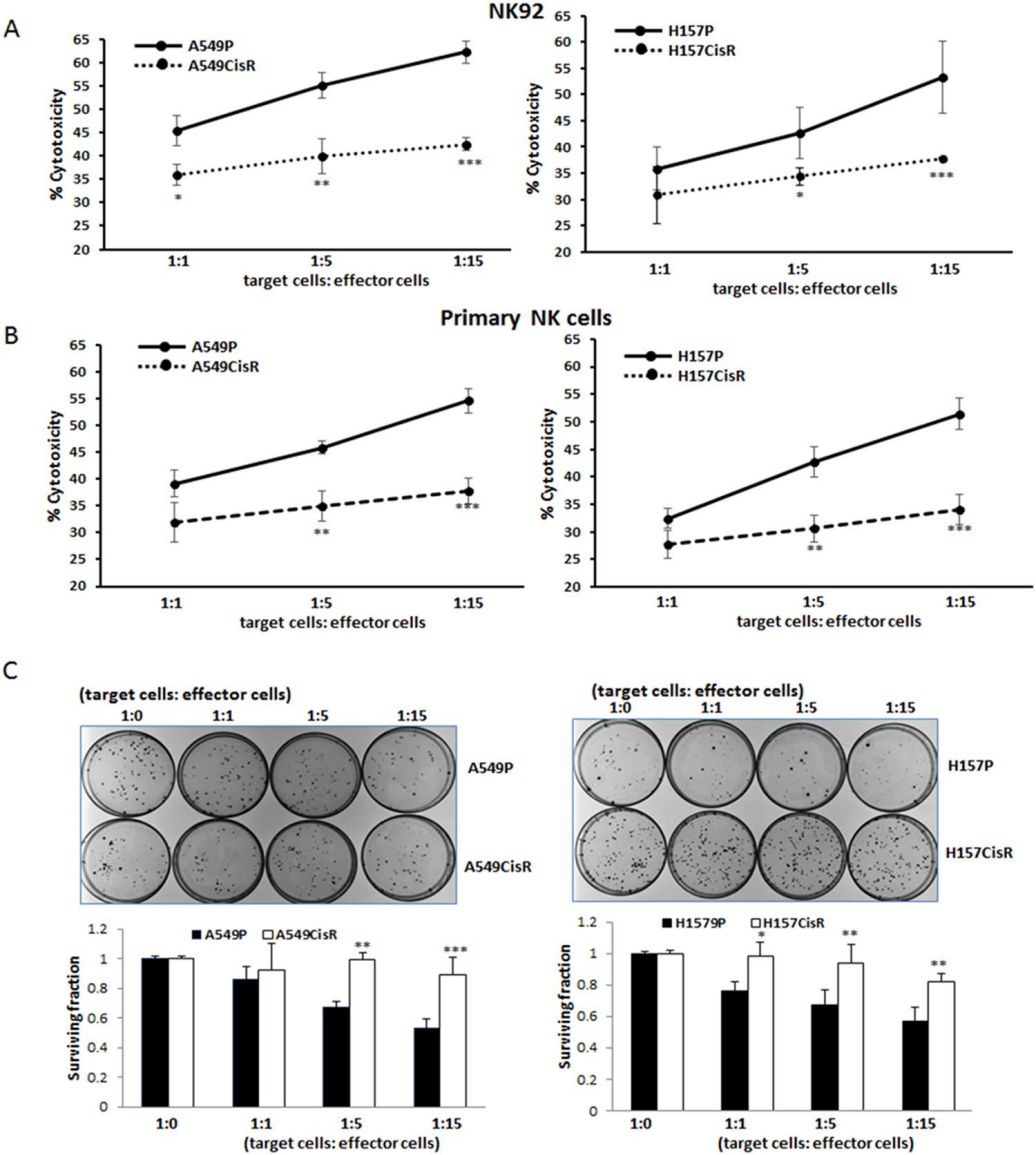
NK cell cytotoxicities to cisplatin-resistant lung cancer cells vs. parental cells. (**A**,**B**) LDH-release based NK cell cytotoxicity tests (**A**), with NK92 cells; (**B**) with primary NK cells). A549P/A549CisR and H157P/H157CisR cells were plated and on the next day either NK 92 cells (**A**) or primary NK cells (**B**) were added at various ratios (in triplicate). Media (50 μl) was collected after 4 hours of tumor cells/NK cells co-culture and the LDH release was measured according to the manufacturer’s instruction. (**C**) Colony formation assay. A549P/A549CisR and H157P/H157CisR cells were plated and NK cells were added similarly as in (**A** and **B**). Media was changed into normal media after 4 hours of tumor cells/NK cells co-culture, survived cells were cultured until colonies become visible, stained with Crystal Violet, and colony numbers were counted under microscope. *p < 0.05, **p < 0.01, ***p < 0.001.

We observed significantly higher resistance to NK92 cell-mediated cytotoxicity (Fig. 1A, left panel, A549CisR cell data; right panel, H157CisR cell data) and to primary NK cell-mediated cytotoxicity (Fig. 1B, left panel, A549CisR cell data; right panel, H157CisR cell data) of cisplatin-resistant cells than the parental cells. Similar findings were observed in the colony formation assay (Fig. 1C). The colonies developed from the survived cells after co-culture with NK cells were visualized. We observed higher colony numbers of A549CisR and H157CisR cells than in parental cells after co-culture with NK92 cells, suggesting lower susceptibility of NK cell-mediated cytotoxicities by cisplatin-resistant cells than parental cells (Fig. 1C, left panel, A549CisR cell data; right panel, H157CisR cell data). Results from both assays suggest that cisplatin-resistant lung cancer cells were more resistant to NK cell-mediated cytotoxic action than parental cells.

### PD-L1 is constitutively up-regulated in cisplatin-resistant lung cancer cells

**Figure 2:**
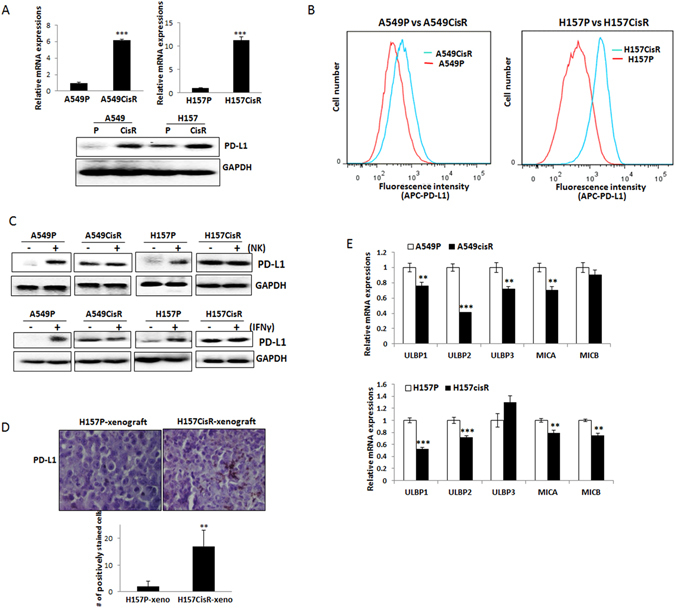
PD-L1/NKG2D ligand expressions in cisplatin-resistant cells vs. parental cells. (**A**) qPCR (upper panel) and Western blot (lower panel) analyses detecting expressions of PD-L1 in A549P/A549CisR and H157P/H157CisR cells. (**B**) Flow cytometric analyses of PD-L1 in A549P/A549CisR and H157P/H157CisR cells. Relative fluorescence stained with APC-conjugated anti-PD-L1 Ab was shown. (**C**) Western blot analysis of PD-L1 in A549P/A549CisR and H157P/H157CisR cells after co-culture with NK cells. Upper panel shows the PD-L1 levels in A549P/A549CisR and H157P/H157CisR cells after co-culture with NK cells for 4 hours. Lower panel shows the PD-L1 levels in A549P/A549CisR and H157P/H157CisR cells after incubation (4 hours) with recombinant ﻿human﻿ IFNγ (rhIFNγ). (**D**) IHC staining of PD-L1 in tumor tissues of H157P and H157CisR-derived xenografts. PD-L1 levels in tumor tissues, derived from H157P cell and H157CisR cell-derived xenografts were examined. Error bars and significance values were obtained by counting positively stained cells in one randomly chosen phase of slides of 3 different stains. Magnification, 20X. (**E**) qPCR analyses of NKG2D ligands in A549P/A549CisR and H157P/H157CisR cells. **p < 0.01, ***p < 0.001.

We investigated the expression of PD-L1 on cisplatin-resistant cells vs. parental cells. We found constitutively up-regulated PD-L1 in A549CisR and H157CisR cells when compared to parental cells (Fig. 2A, upper panel, mRNA level; lower panel, protein level). Flow cytometric analysis also found higher levels of cell surface PD-L1 expression in cisplatin-resistant cells than in parental cells (Fig. 2B).

We then investigated whether the up-regulated PD-L1 level in cisplatin-resistant cells was due to the IFNγ secreted by NK cells, as it has been previously reported that the IFNγ secreted by NK cells could induce PD-L1^27, 28^. A549P/A549CisR and H157P/H157CisR cells were co-cultured with NK cells and the PD-L1 protein expression levels in tumor cells were analyzed in comparison with the basal levels in tumor cells without co-culture. Figure 2C (upper panel) showed an increase of PD-L1 in A549P and H157P cells upon co-culture with NK cells, but such an increase was not observed in A549CisR and H157CisR cells. We also investigated the PD-L1 induction upon direct treatment of tumor cells with recombinant human IFNγ (r﻿hIFNγ). Similar to data shown in the co-culture experiment with NK cells, the PD-L1 level in parental cells were induced by rhIFNγ treatment, but such an increase was not observed in cisplatin-resistant cells (Fig. 2C, lower panel).

We then sought to confirm the *in vitro* findings in the tumors in *vivo*. We investigated PD-L1 expression in tumor tissues of H157CisR cell- derived xenografts vs. H157P cell-derived human tumor xenografts. Orthotopic xenografts were developed by injection of luciferase-tagged H157P and H157CisR cells in the chest cavity. The immunohistochemical (IHC) staining results of excised tumor tissues showed higher numbers of PD-L1 positive cells in tumor tissues of H157CisR-xenografts than in H157P-xenografts (Fig. 2D). In all, our data suggested that higher levels of PD-L1 were constitutively expressed in cisplatin-resistant lung cancer cells and might play a critical role in the resistance to NK cell cytotoxicity.

### NKG2D ligands were reduced in cisplatin-resistant cells compared to parental cells

We compared the levels of NKG2D ligands in tumor cells, which have also been suggested to be important in the interaction of NK cells with tumor cells^17^. When mRNA levels of five well-known NKG2D ligands ULBP1, ULBP2, ULBP3, MICA, and MICB were analyzed in A549P/A549CisR vs. H157P/H157CisR cells, we found most of these ligands (except ULBP3 in H157 cells) were down-regulated in A549CisR and H157CisR cells compared with parental cells (Fig. 2E).

### PD-1 expression in NK cells was induced when incubated with tumor cells, and showed higher induction after incubation with cisplatin-resistant cells than with parental cells

**Figure 3:**
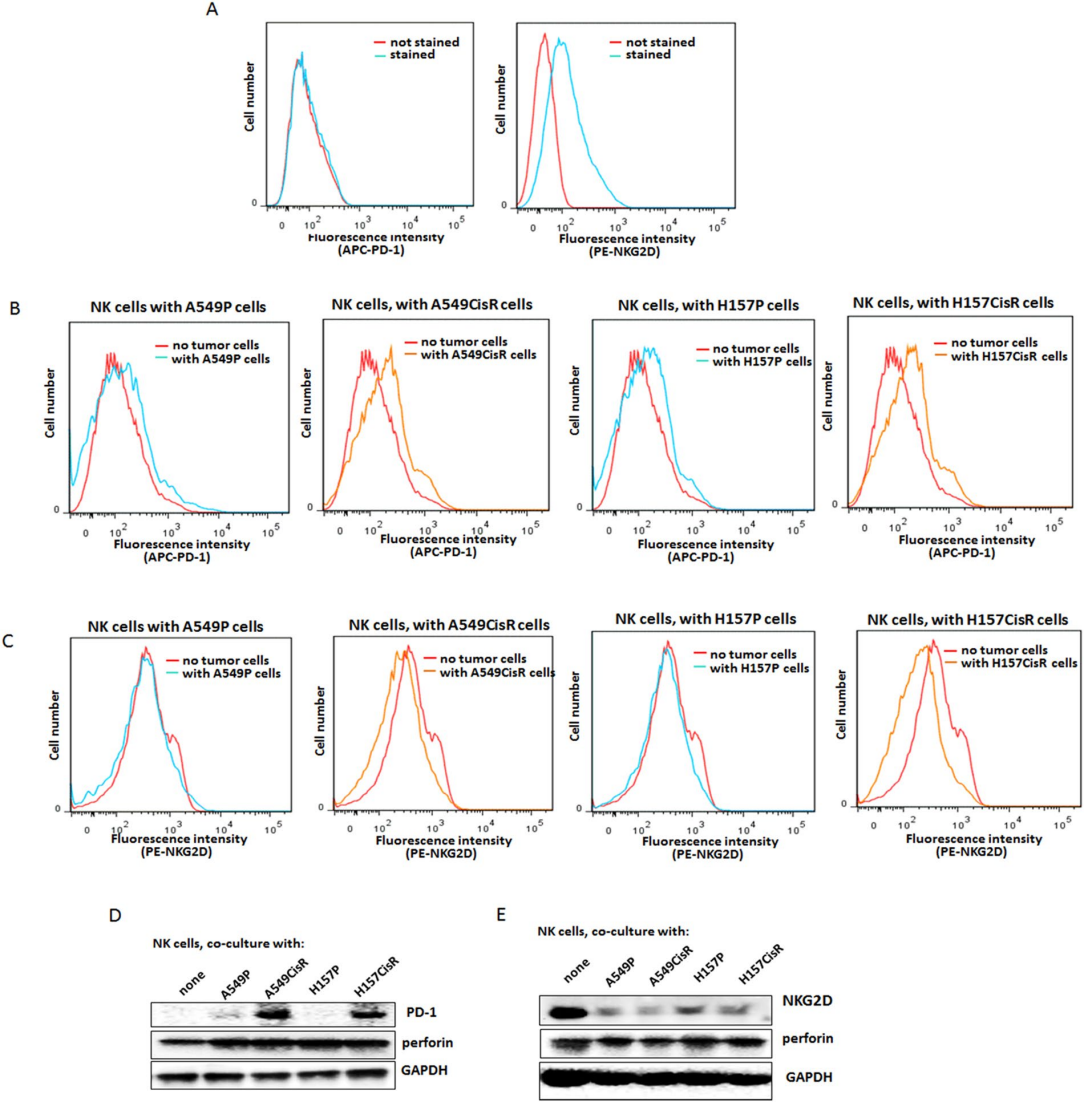
Expression of PD-1/NKG2D in NK cells, with or without co-culture with tumor cells. (**A**) Flow cytometric analyses of NKG2D and PD-1 in NK cells. Primary NK cells were stained with PE-NKG2D or APC-PD-1 and positive staining was analyzed. (**B**) Flow cytometric analyses of PD-1 on NK cells, after co-culture with tumor cells (6 hours of incubation). Primary NK cells were added into tumor cells (1:1 ratio, tumor cells:NK cells) and collected after 6 hours of incubation. PD-1 level in the collected NK cells were analyzed in flow cytometric analysis (using APC-PD-1 Ab). (**C**) Flow cytometric analyses of NKG2D on NK cells, after co-culture with tumor cells (6 hours of incubation). (**D**) Western blot analyses detecting PD-1 levels in NK cells after co-culture with tumor cells. NK92 cells were used in these tests. (**E**) Western blot analyses of NKG2D levels in NK92 cells when co-cultured with tumor cells. **p < 0.01, ***p < 0.001.

We investigated the PD-1 level in NK cells as it was reported that PD-1 is expressed at a high level in T cells^29^, but little is known regarding the PD-1 level on NK cells. In flow cytometric analyses using primary NK cells, we observed positive NKG2D staining, but no positive PD-1 staining (Fig. 3A). We then speculated PD-1 expression might be induced when NK cells are exposed to tumor cells to allow for high PD-L1/PD-1 interaction between tumor cells and NK cells. To test this hypothesis, we analyzed the PD-1 levels in NK cells in flow cytometric and Western blot analyses after co-culture with tumor cells. In flow cytometric analyses, we found the PD-1 levels in primary NK cells were increased upon incubation with tumor cells, and the induction level was more significant when incubated with cisplatin-resistant cells than with parental cells (Fig. 3B). We also detected increased PD-1 levels in NK92 cells in Western blot analyses upon co-culture with tumor cells with an almost undetectable PD-1 level. Consistently, we found the induced PD-1 level was markedly higher when incubated with A549CisR and H157CisR cells than when incubated with A549P and H157P cells (Fig. 3D). These findings support our hypothesis that a higher PD-L1/PD-1 interaction exists between cisplatin-resistant cells and NK cells than between parental cells and NK cells.

On the other hand, we detected a reduced NKG2D level in NK92 cells when incubated with tumor cells and higher reduction was observed when incubated with A549CisR and H157CisR cells than with A549P and H157P cells. Figure 3C shows flow cytometric analysis results analyzing the surface NKG2D on NK cells, and Fig. 3E shows the Western blot analysis data.

These results suggest that the induced PD-1 level with reduced NKG2D levels in NK cells may trigger inhibitory effects on NK cell cytotoxicity in cisplatin-resistant cells.

### Inhibiting PD-L1/PD-1 axis increased susceptibility of cisplatin-resistant cells to NK cell cytotoxicity

**Figure 4:**
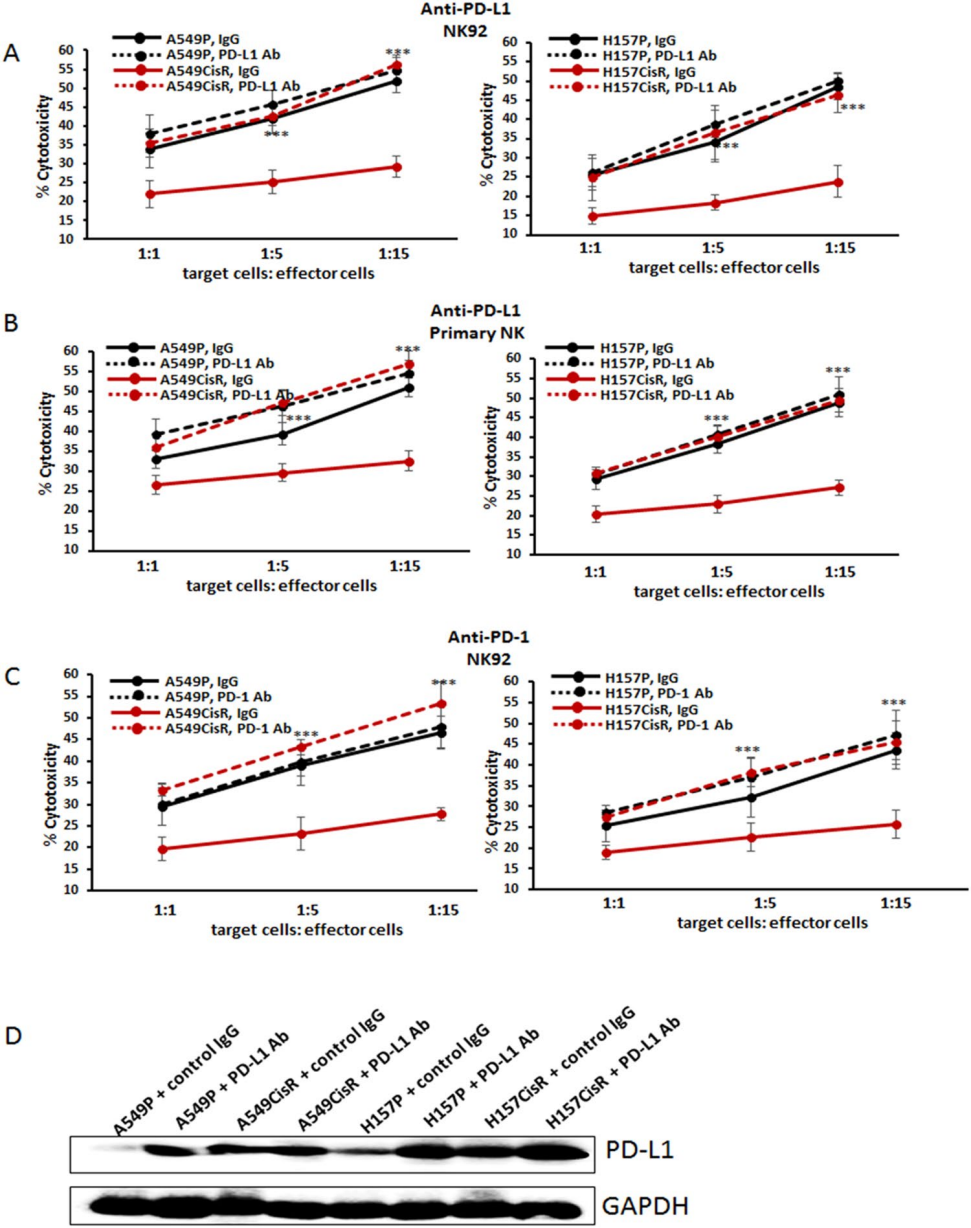
Effects of neutralizing Abs of PD-L1 or PD-1 on the susceptibilities of cisplatin-resistant lung cancer cells to NK cell cytotoxicity. (**A**,**B**) NK cytotoxicity tests to A549P/A549CisR and H157P/H157CisR cells upon addition of anti-PD-L1 Ab to tumor cell/NK cell co-culture. (**A** with NK92 cell line; **B** with primary NK cells) (**C**). NK cell cytotoxicity tests to A549P/A549CisR and H157P/H157CisR cells upon addition of anti-PD-1 Ab to tumor cell/NK cell co-culture (with NK92 cells). (**D**) Western blot analyses showing PD-L1 levels in A549P/A549CisR and H157P/H157CisR cells after incubation with either control IgG or PD-L1 Ab. *p < 0.05, **p < 0.01, ***p < 0.001.

We then investigated whether blocking the PD-L1/PD-1 axis might increase susceptibility of cisplatin-resistant cells to NK cell cytotoxic action. NK cell cytotoxicities to A549P/A549CisR and H157P/H157CisR cells were tested after adding the neutralizing Ab of PD-L1 (or control IgG) into tumor/NK cell co-cultures. As shown in Fig. 4A (NK92 cell data) and Fig. 4B (primary NK cell data), the addition of the PD-L1 antibody significantly increased susceptibility of A549CisR and H157CisR cells to NK cell cytotoxicity, but the susceptibility of parental A549P and H157P cells to NK cell cytotoxicity were not significantly influenced. Furthermore, we observed increased susceptibility of cisplatin-resistant cells to NK92 cell cytotoxicity upon addition of PD-1 Ab, but the susceptibility of parental cells remained unchanged (Fig. 4C). Taken together, results in Fig. 4 supported that disrupting the PD-L1/PD-1 interaction enhanced the susceptibility of cisplatin-resistant cells to NK cell cytotoxicity.

We tested whether addition of PD-L1 Ab alters intracellular PD-L1 level in tumor cells. We found the PD-L1 Ab treatment did not reduce intracellular PD-L1 level in parental and cisplatin-resistant cells. Instead, we observed slightly increased expression of PD-L1 after PD-L1 Ab treatment (Fig. 4D), suggesting that addition of PD-L1 Ab acted via disruption of PD-L1/PD-1 interaction, but did not affect intracellular PD-L1 level in cisplatin-resistant cells.

### JAK/STAT/MAPK/Erk signaling pathways were activated in cisplatin-resistant cells compared to parental cells, among which, MEK/Erk signaling was most responsible for the constitutive expression of PD-L1 in cisplatin-resistant cells

**Figure 5:**
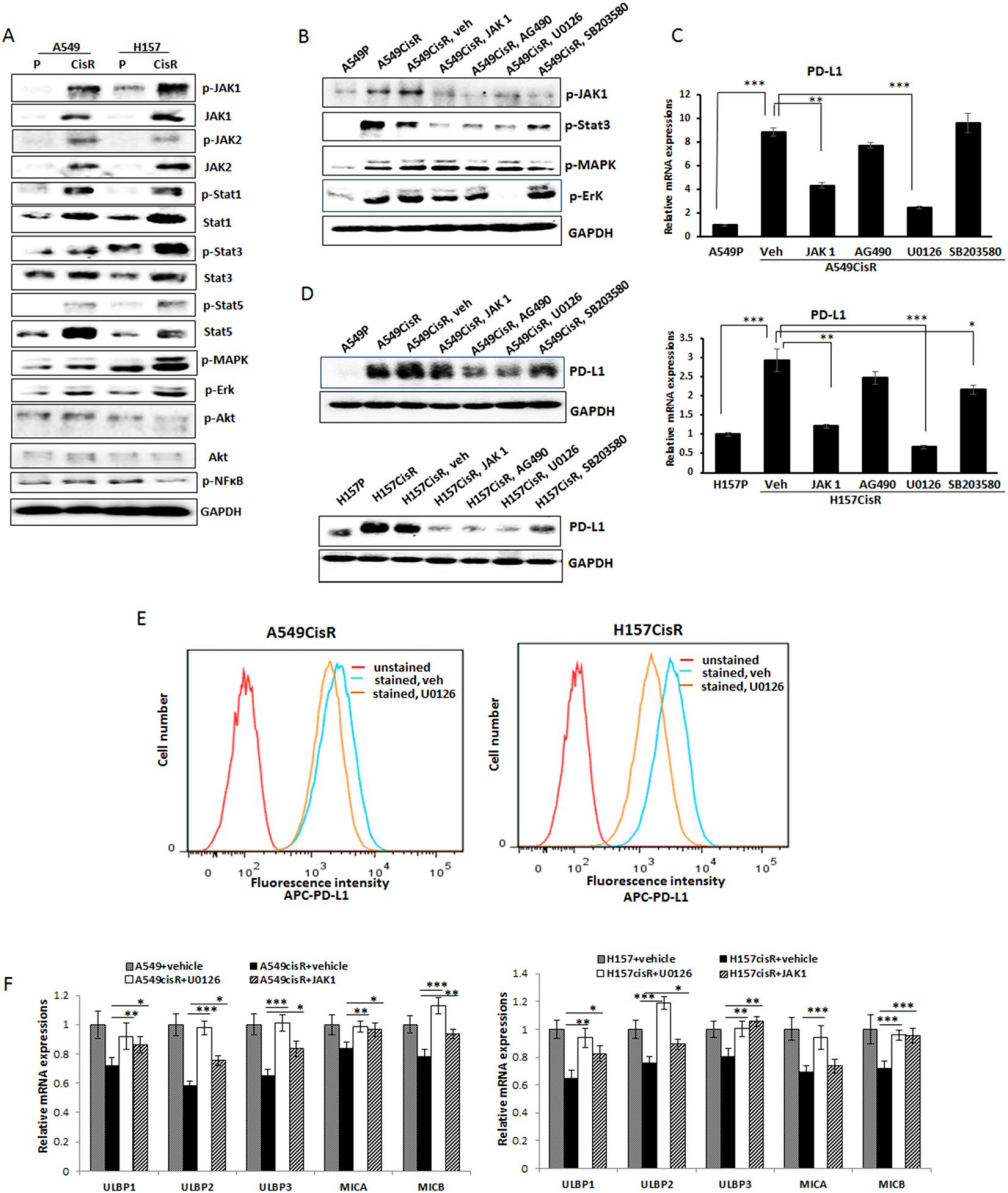
Effects of signaling pathway inhibition on PD-L1 levels in A549CisR and H157CisR cells. (**A**) Western blot analyses showing the expression/activation of several signaling molecules in parental and cisplatin-resistant cells. (**B**) Western blot analyses showing inhibition of each pathway in A549CisR cells upon inhibitor treatment. (**C**,**D**) PD-L1 levels in A549CisR and H157CisR cells upon treatment with inhibitors of indicated signaling pathways using qPCR analysis (**C**) and Western blot analysis (**D**). (**E**) Flow cytometric analyses of PD-L1 in cisplatin-resistant lung cancer cells. A549CisR and H157CisR cells were treated with U0126 (6 hours) (vehicle treated one as control) and positive stained APC-PD-L1 levels in these cells were analyzed. (**F**) NKG2D ligand expression on the A549CisR and H157CisR cells vs. parental cells. qPCR analyses show the recovery of NKG2D ligands upon addition of inhibitors of MEK/Erk and JAK signaling pathways. *p < 0.05, **p < 0.01, ***p < 0.001.

To reveal the potential signaling pathways responsible for the constitutive expression of PD-L1 in cisplatin-resistant cells, we compared the activation of several candidate signaling pathways in A549P/A549CisR and H157P/H157CisR cells that were reported to be involved in the up-regulation of PD-L1 in several types of tumors. These include JAK1/2^27, 30^, Stat 1^27^, Stat3^31, 32^, Stat5^33, 34^, NFκB^35^, MEK/Erk^36, 37^, PI3K/Akt^38, 39^, and MAPK^40^ pathways. We found almost all of these molecules/signaling pathways were up-regulated/activated in A549CisR and H157CisR cells when compared to A549P and H157P cells, while we found no significant activation of NFκB and Akt pathways (Fig. 5A). We then tested whether treating cisplatin-resistant cells with inhibitors of candidate signaling pathways^41^ could lower the constitutively expressed PD-L1 (inhibition of each pathway upon inhibitor treatments is shown in Fig. 5B). As shown in Fig. 5C (qPCR test results) and **5D** (Western blot results), treatment with inhibitors of JAK (JAK inhibitor 1), JAK/Stat (AG490), MEK/Erk (U0126), and MAPK (SB203580) signaling pathways had all decreased the PD-L1 level in cisplatin-resistant cells, but the most significant effect was observed with the MEK/Erk inhibitor (U0126). These results suggest that the MEK/Erk signaling pathways may contribute most significantly to the constitutive up-regulation of PD-L1 in cisplatin-resistant cells. Decreased PD-L1 levels in cisplatin-resistant cells upon MEK/Erk inhibition were also confirmed in flow cytometric analyses (Fig. 5E).

### MEK/Erk inhibitor treatment recovered the reduced NKG2D ligands in cisplatin-resistant cells

We then investigated whether inhibition of MEK/Erk signaling could recover the reduced NKG2D ligands in cisplatin-resistant cells. Since the JAK signaling also exhibited some effects in reducing PD-L1 levels (Fig. 5C and D), we added the JAK inhibitor in this experiment in parallel. We found the NKG2D levels were recovered upon addition of the MEK/Erk inhibitor (Fig. 5F), suggesting that the MEK/Erk signaling was important not only in up-regulation of PD-L1 in cisplatin-resistant cells, but also in triggering reduction of NKG2D ligands in these cells. The JAK inhibitor also showed some effects, but its effect was not to the extent as the MEK/Erk inhibitor (data not shown).

### Combined treatment of the MEK/Erk inhibitor and PD-L1 antibody further elevated the NK cytotoxic action of NK cells to cisplatin-resistant cells

**Figure 6:**
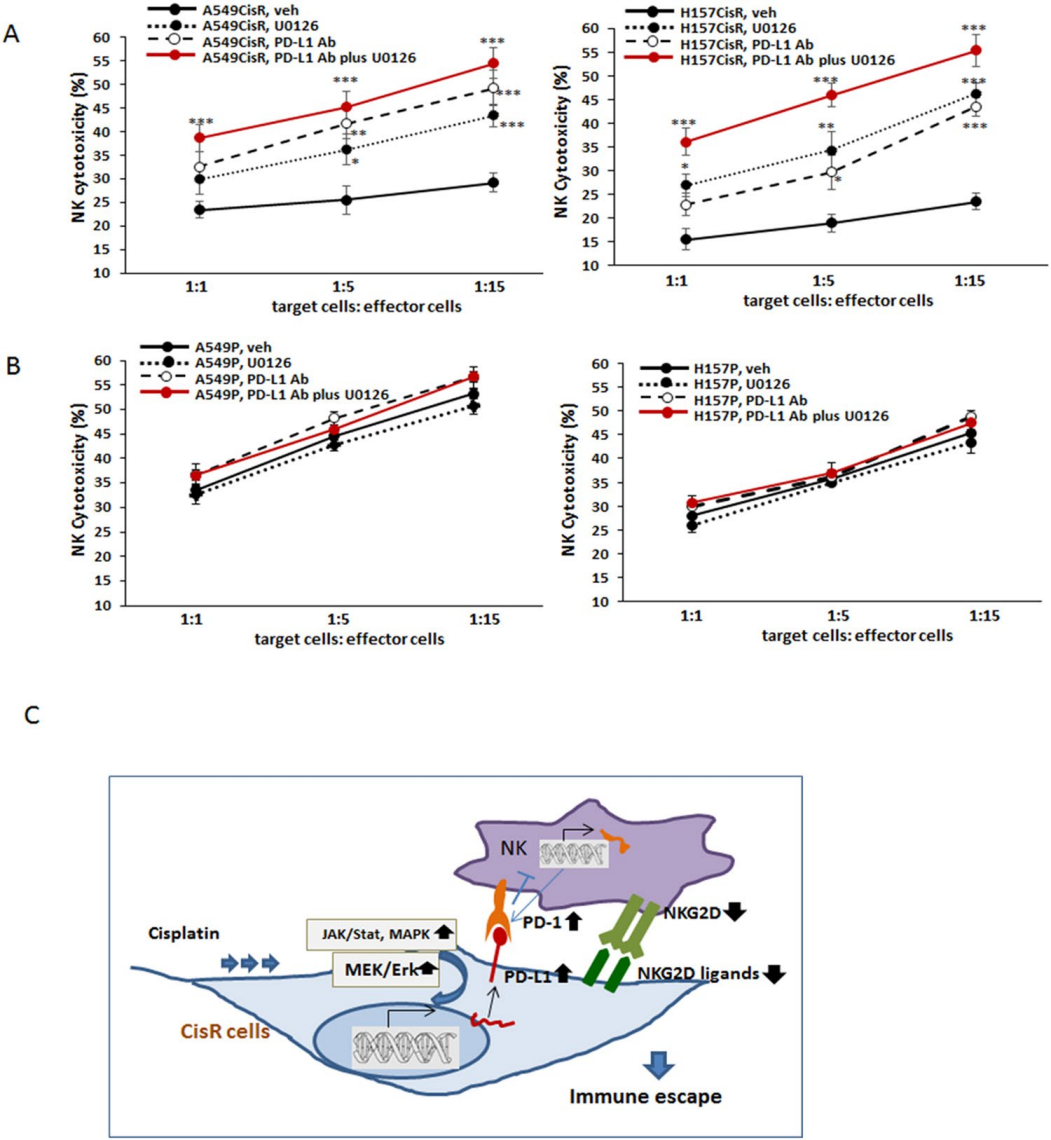
Combined effects of inhibitor of MEK/Erk signaling pathway and PD-L1 Ab on NK cytotoxicity. The susceptibility of cisplatin-resistant lung cancer cells to cytotoxic action of primary NK cells was enhanced when the inhibitor of MEK/Erk signaling pathway and PD-L1 Ab were both present. (**A**,**B**) (**A**) NK cytotoxicity tests (primary NK cells) of A549CisR and H157CisR cells. (**B**) A549P and H157P cells in the presence of PD-L1Ab alone, U0126 alone, vs. both present in the co-culture of tumor cells/primary NK cells. (**C)** A cartoon depicting tumor cell and NK cell interactions through PD-L1/PD-1 and NKG2D ligands/NKG2D axis. *p < 0.05, **p < 0.01, ***p < 0.001.

As we found that the MEK/Erk signaling was most critical in constitutive expression PD-L1 and the down-regulation of NKG2D ligands in cisplatin-resistant cells, we next tested whether the addition of the MEK/Erk inhibitor to the primary NK/tumor cell co-culture might enhance the PD-L1 Ab effect on increasing the susceptibility of cisplatin-resistant cells to NK cell action. We found that combined use of the PD-L1 Ab and the inhibitor of the MEK/Erk signaling showed significantly enhanced susceptibility of cisplatin-resistant cells to primary NK cell cytotoxicity when compared to the effect of either PD-L1 Ab or the inhibitor alone (Fig. 6A, left panel, A549CisR cell data, right panel, H157CisR cell data). In contrast, such an effect was not observed in parental cells (Fig. 6B, left panel, A549P cell data, right panel, H157P cell data).

In Fig. 6C, we demonstrated our hypothesis in a cartoon describing the interaction of tumor cells and NK cells through PD-L1/PD-1 axis, NKG2D/NKG2D ligands, and the enhancement by Jak/Stat, MAPK, and MEK/Erk pathways.

## Discussion

While immunotherapy showed promise in treating lung cancer patients who failed chemotherapy, developing immune escape of tumor cells remains an obstacle of effective immunotherapy^42, 43^. Previous research has primarily been focused on immune escape of tumor cells from T cell and dendritic cell-mediated immunity^7, 8^, and the reports on immune escape from NK cell cytotoxic action are limited. In this study, we studied immune escape of cisplatin-resistant lung cancer cells from NK cell cytotoxicity. Similarly to the reports showing the correlation of immune escape of tumor cells from the T cell/dendritic cell-mediated immunity with high PD-L1 levels in tumor cells^8, 9^, we observed the correlation of immune escape from NK cell cytotoxicity with high PD-L1 levels in tumor cells. This result is consistent with the report by Belluci *et al*.^27^ who also suggested that immune escape of hematopoietic tumor cells from NK cell action may be due to high PD-L1 levels in tumor cells.

When we investigated PD-L1 level in lung cancer cell lines, we found the PD-L1 level varies in cell lines. We detected almost no PD-L1 in A549P cells, which was consistent with the result by Chen *et al*.^37^, but detected low expressions of PD-L1 in H157P cells. However, in both cell lines, markedly increased levels of PD-L1 were detected in cisplatin-resistant sublines of these cells, which was consistent with the previous reports showing the PD-L1 increase in chemoresistant cancer cells^35, 42^. However, we could not observe a further increase in PD-L1 levels in cisplatin-resistant lung cancer cells upon IFNγ treatment, probably due to the fact that PD-L1 levels in cisplatin-resistant lung cancer cells were high or the signaling pathways responsible for the up-regulation of PD-L1 were already highly activated in these cells.

High PD-1 levels in T cells and its role in immune evasion was reported^29^. Saito *et al*.^44^ showed that the PD-1 expression on CD4+ and CD8+ T cells of gastric cancer patients was significantly higher than that of normal controls, and thus suggested that the increased PD-1 expression in T cells was associated with the immune evasion of tumor cells. It was shown that the expression of PD-1 on CD4+ T cells in peripheral blood was associated with poorer clinical outcomes in NSCLC^45^.

However, limited information is available regarding PD-1 expression in NK cells. Induction of PD-1 level in NK cells after viral infection has been reported. Pesce *et al*.^46^ found that one subset of NK cells expressed high PD-1, and found this population was increased in patients with ovarian carcinoma. On the other hand, Guo *et al*.^47^ reported that the PD-1 level in freshly isolated NK cells was undetectable, but was induced when expanded in culture. Benson *et al*.^10^ reported that the PD-L1/PD-1 axis is important in modulating the NK cell action to multiple myeloma cells, supporting the PD-1 expression on NK cells.

We noted an undetectable basal PD-1 level on NK cells, but found a markedly increased PD-1 level on NK cells after incubation with cisplatin-resistant lung cancer cells, not with parental cells. The discovery of showing the PD-1 induction in NK cells after incubation with cisplatin-resistant lung cancer cells seems clinically significant as it confirms the existence of high PD-L1/PD-1 interaction between cisplatin-resistant lung cancer cells and NK cells. This also explains why we observed effects of blocking the PD-L1/PD-1 checkpoint on increasing NK cytotoxicity to cisplatin-resistant cells, but not to parental cells. We hypothesized that the induction of PD-1 levels in NK cells might be one of the mechanisms by which cisplatin-resistant lung cancer cells evade from the NK cell-mediated immune reaction.

The NKG2D/NKG2D ligand axis was also reported to be important in interaction between tumor cells and NK cells^48^. While the PD-L1/PD-1 axis acts as an inhibitory signal for NK cell action, the NKG2D ligands/NKG2D interaction is known as an activation signal for their interaction. The importance of increased NKG2D ligand in enhancing NK cell cytotoxicity to tumor cells was also suggested by Shi *et al*.^24^. They showed that increasing the level of one NKG2D ligand, ULBP2, enhanced NK cell cytotoxicity to hepatocarcinoma cells. In addition to ULBP2, five ligands that we studied were all down-regulated in cisplatin-resistant lung cancer cells.

In flow cytometric and Western blot analyses, we found the NKG2D level in NK cells was also decreased upon incubation with cisplatin-resistant lung cancer cells, suggesting that the reduction of both the NKG2D ligand level in tumor cells and the NKG2D level in NK cells will lead to the reduced NKG2D ligand/NKG2D interaction, which may result in developing the immune escape of cisplatin-resistant lung cancer cells from the NK cell action. Further investigation is necessary to prove whether NK cell cytotoxicity to cisplatin-resistant lung cancer cells increases when blocking the NKG2D ligands/NKG2D interaction. In addition, exploring molecular mechanisms by which tumor cell (especially cisplatin-resistant cells) incubation alters PD-1/NKG2D levels in NK cells is necessary.

In mechanism studies, we found most significant effect of MEK/Erk signaling inhibition on lowering the constitutively expressed PD-L1 level while recovering NKG2D ligand level in cisplatin-resistant lung cancer cells. The second candidate was the inhibition of JAK signaling pathway. However, when we tested the effect of JAK inhibitor on increasing the susceptibility of cisplatin-resistant cells to NK cell cytotoxicity, no significant effect was observed (data not shown). We indeed observed that the MEK/Erk inhibition on suppressing the PD-L1 level (both total and surface level) on cisplatin-resistant lung cancer cells. Therefore, we observed the MEK/Erk inhibition on directly enhancing the NK cell cytotoxicity to cisplatin-resistant lung cancer cells. While the PD-L1 Ab effect is through inhibition of the PD-L1/PD-1 interaction, use of the MEK/Erk inhibitor may trigger down-regulation of PD-L1 and recovery of NKG2D ligands simultaneously, thus possibly a more effective strategy that can apply to future immunotherapeutic approaches. Animal studies will be necessary to test the effect of the MEK/Erk inhibitor. We suggested the effects of PD-L1 Ab or MEK/Erk inhibition on directly enhancing the NK cell cytotoxicity to cisplatin-resistant lung cancer cells, but Benson *et al*.^10^ suggested that the PD-L1Ab effect may be through affecting NK cell trafficking and immune complex formation with tumor cells.

More importantly, we found that the combined use of PD-L1 Ab and the MEK/Erk inhibitor together significantly enhanced the NK cytotoxic effect to cisplatin-resistant lung cancer cells than using the PD-L1 Ab or the inhibitor alone. The combined use of the MEK inhibitor and PD-L1Ab may have the potential for clinical applications, especially in targeting lung cancer patients at the cisplatin-resistant stage.

The MEK inhibitors are in clinical trials to treat many types of cancers, including lung cancer. The MEK inhibitor CI-1040 has been used in clinical trials, but demonstrated insufficient antitumor activity^49, 50^. Meanwhile PD 0325901, a second generation MEK inhibitor, has recently entered clinical development and has showed significantly improved pharmacologic and pharmaceutical properties^50^. Though they have showed promising effects, ERK inhibitors are still in clinical development due to their toxicity^51^.

Whether PD-L1 plays a critical role in developing chemoresistance is not clear. A clinical trial of the PD-L1 blocking therapy using Nivolumab (PD-L1 Ab) for the treatment of metastatic lung cancer with progression before or after docetaxel or platinum chemotherapy has been performed and showed restoration of antitumor immunity^52, 53^. Recently, it was suggested that the activation of PD-1/PD-L1 axis led tumor cells resistant to conventional drugs^42^ and Yan *et al*.^54^ found that elevated cellular PD-L1/PD-1 expression confers acquired resistance to cisplatin in small cell lung cancer cells. However, our studies showed that the PD-L1 antibody treatment did not increase cisplatin-sensitivity of cisplatin-resistant lung cancer cells (data not shown), suggesting that PD-L1 increase might not be directly involved in the development of cisplatin-resistance. Further investigation will be necessary to make a conclusion.

## Methods and Materials

### Cell culture

A549 (CLL-185^TM^) and H157 (CRL-5802^TM^) cell lines were purchased from the American Type Culture Collection (ATCC) and cultured in RPMI 1640 containing 10% FBS. All cells were maintained in a humidified 5% CO_2_ environment at 37 °C. NK92 cell line (CRL2407^TM^) was also purchased from ATCC and cultured in α-MEM media containing sodium bicarbonate (Sigma, M4655), IL-2 (100 units/ml) (Peprotech, 200-02), inositol (0.2 mM, Sigma), 2-mercaptoethanol (0.1 mM), folic acid (0.02 mM), 12.5% horse serum (Sigma) and 12.5% FBS (Hyclone). For inhibitor studies, JAK inhibitor 1 (5 μM) (Calbiochem, CAS457081-03-7), AG490 (5 µM) (Sigma, T3404), U0126 (10 µM) (Cell Signaling, 9903), and SB203580 (10 µM) (Sigma, 559387) that inhibit JAK, JAK/Stat3, MEK/Erk, and MAPK pathways, respectively, were added into the co-culture of tumor cells/NK cells.

### Development of cisplatin-resistant cell lines

Parental A549 (A549P) and H157 (H157P) cells were continuously treated with a gradually increased concentration of cisplatin for 6 months according to the method described by Barr *et al*.^55^. Briefly, cells were treated with 1 µM cisplatin for 72 hours and allowed to recover for the following 72 hours. After repeating one more cycle at 1 µM cisplatin concentration, the cells were then treated with 2 µM cisplatin in the following two cycles. This procedure was repeated with increasing cisplatin concentrations up to 30 µM. During the cisplatin-resistance induction procedure, the IC_50_ values of every 5 passages were determined in cisplatin-cytotoxicity tests and compared with those of the parental cells. The treatment continued until the increased IC_50_ value remained unchanged. The cisplatin-resistant cell lines obtained by this method were maintained in growth media containing 10 µM cisplatin.

### Cisplatin-cytotoxicity test

Cisplatin-cytotoxicity was analyzed by MTT (3-[4,5-dimethylthiazol-2-yl]-2,5-diphenyltetrazolium bromide, 5 mg/ml, Sigma, USA) assay. Cells (A549P/A549CisR and H157P/H157CisR) were seeded on 96-well plates (5 × 10^3^ cells/well) and treated with various concentrations of cisplatin for 48 hours. MTT test was then performed and absorbance at 490 nm was measured. Cell viability was calculated using the formula: OD sample/OD blank control × 100. Triplicate experiments were performed and average values with mean ± SEM were represented.

### NK cells

We had two NK cell sources for the NK-cytotoxicity tests: NK 92 and primary NK cells. The established NK cell line NK92 was purchased from ATCC (CRL2407^TM^). The primary NK cells were isolated and purified from peripheral blood mononuclear cells (PBMCs) of healthy donors using NK cell isolation kit (Miltenyi Biotec, 130-092-657) according to manufacturer’s protocol. After the isolation, isolated cells were maintained in IL-2 containing NK cell media. The purity of isolated cells (CD56+ CD3−) was confirmed by flow cytometric analyses using anti-CD56-PE (e-Bioscience, 12-0267-41) and anti-CD3-Cy7 (BioLegend, 300429) antibodies.

### NK cytotoxicity tests (LDH release-based)

NK cell cytotoxicity against tumor cells (A549P/A549CisR and H157P/H157CisR) was analyzed using a lactate dehydrogenase (LDH) release assay. Cells (2,500 to 5,000 cells) were plated, and on the next day NK cells were added at various ratios (1:1, 1:5, and 1:15, target cells: effector cells) (all samples in triplicate). After 4 hours of co-culture, an aliquot of 50 μl media was used in LDH cytotoxic assay using the LDH cytotoxic assay kit (Thermo Fisher Scientific, 88954). The value of corrected experimental LDH release was calculated by subtracting the value of spontaneous LDH release from effector cells at corresponding dilutions. NK cytotoxicity was defined as %Cytotoxicity = (Experimental value − Effector Cells Spontaneous Control − Target Cells Spontaneous Control)/(Target Cell Maximum Control − Target Cells Spontaneous Control) × 100.

### Colony formation assay

Cells (2,500 to 5,000 cells) were plated and on the next day after NK cells were added at various ratios (1:1, 1:5, and 1:15, target cells: effector cells with all samples in triplicate). After 4 hours of co-culture, NK cells were removed and fresh media was added into tumor cells. After 10 days of culture, colonies formed were visualized by crystal violet staining and the colony numbers were counted under a microscope.

### Flow cytometric analysis

A549P/A549CisR and H157P/H157CisR cells were stained with APC-PD-L1 Ab (BioLegend, 329707) (5 μl/10^6^ cells) (unstained cells as control) while NK cells were stained with PE-NKG2D Ab (BioLegend, 320805) or APC-PD-1 Ab (BioLegend, 329907), and the fluorescence was detected using the Canto II system (Becton-Dickinson).

### *In vivo* xenograft studies

The luciferase tagged H157P and H157CisR cells (1 × 10^6^) obtained by transfection of luciferase reporter gene and the selection procedure. These cells were orthotopically injected (1 × 10^6^ cells in media with Matrigel, 1:1 ratio in volume) into 8-week old female nude mice (NCI) (n = 6 per group). Tumor development was monitored once a week and the changes in tumor volume assessed using the *In Vivo* Imaging System (IVIS). All animal studies were performed under the supervision and guidelines of the University of Rochester Medical Center’s Animal Care and Use Committee. The experimental protocol was approved by the University of Rochester, University Committee on Animal Resources (Protocol number: 101285/2008-092).

### Histology and immunohistochemistry

Tumor tissues obtained from xenografts were fixed in 10% (v/v) formaldehyde in PBS, embedded in paraffin, and cut into 5-µm sections. Tumor tissue sections were deparaffinized in xylene solution, rehydrated, and immunostained with the IHC kit (Santa Cruz, SC2018) and stained for PD-L1 using PD-L1 antibody (R&D, MAB1086). After staining, tissues were counterstained by Hematoxylin. After staining, three areas were randomly selected from slides of three different stains by an investigator not involved in this study, and positive stained cell numbers were obtained.

### RNA extraction and quantitative real-time PCR (qPCR) analysis

Total RNA (1 µg) was subjected to reverse transcription using Superscript III transcriptase (Invitrogen). qPCR was conducted using the appropriate primers and a Bio-Rad CFX96 system with SYBR green to determine the mRNA expression levels of genes of interest. Expression levels were normalized to GAPDH mRNA level.

### Western Blot analysis

Cells were lysed in RIPA buffer (50 mM Tris-Cl at pH 7.5, 150 mM NaCl, 1% NP-40, 0.5% sodium deoxycholate, 1 mM EDTA, 1 μg/mL leupeptin, 1 μg/mL aprotinin, 0.2 mM PMSF). Proteins (20–40 µg) were separated on 8–10% SDS/PAGE gel and then transferred onto PVDF membranes (Millipore, IPVH00010). After the blocking procedure, membranes were incubated with primary antibodies (1:1000) and HRP-conjugated secondary antibodies (1:5000), and visualized in Imager (Bio-Rad) using ECL system (Thermo Fisher Scientific, 34095). Antibodies used were: PD-L1 (R&D, MAB1086), NKG2D (R&D, MAB139), PD-1 (R&D, MAB1086), p-JAK1 (Y1022, Assay Biotech, A7125), p-JAK2 (Y1007 + 1008, Abbomax, 601–670), JAK1 (Abgent, AP20699a), JAK2 (Abgent, AP20700c), p-Stat1 (S727, Millipore, 07–714), Stat1 (Abgent, AP19835Bb), p-Stat3 (Y705, Abcam, ab76315), Stat3 (Abcam, ab5073), p-Stat5 (Y694, Abcam, ab32364), Stat5 (Abcam, ab16276), p-MAPK (Cell Signaling, 9101 S), p-Erk (Cell Signaling, 4695), p-Akt (S473, Cell Signaling, 9271), p-NFκB (S536, Abcam ab86299), and GAPDH (Cell Signaling, 2118 S).

### Statistics

The data were presented as the mean ± SEM. Differences in mean values between two groups were analyzed by two-tailed Student’s *t* test. *p* ≤ 0.05 was considered statistically significant.
